# Application of stem cells alone and in combination with low-level laser therapy for sciatic nerve repair in rats

**DOI:** 10.1186/s13018-025-05455-2

**Published:** 2025-01-18

**Authors:** Mohsen Shalalvand, Hamidreza Mahaseni Aghdam, Ahmad Asghari, Siamak Nehzat, Fatemeh Shahsavari, Rojin Ardalani

**Affiliations:** 1 Department of Oral and Maxillofacial Radiology, School of Dentistry, Babol University of Medical Sciences, Babol, Iran; 2 Department of Oral and Maxillofacial Surgery and Implant Research Center, Faculty of Dentistry, Tehran Medical Sciences, Islamic Azad University, Tehran, 19395/1495 Iran; 3 Department of Clinical Science, Science and Research Branch, Islamic Azad University, Tehran, 1477893855 Iran; 4 Department of Clinical Periodontology, Faculty of Dentistry, Tehran Medical Sciences, Islamic Azad University, Tehran, 19395/1495 Iran; 5 Department of Oral and Maxillofacial Pathology, Faculty of Dentistry, Tehran Medical Sciences, Islamic Azad University, Tehran, 19395/1495 Iran; 6 Department of Oral and Maxillofacial Radiology, School of Dentistry, Isfahan (Khorasgan) Branch, Islamic Azad University, Isfahan, Iran

**Keywords:** Mesenchymal stem cells, Stem cells, Sciatic nerve repair, Low-level laser therapy, Nerve regeneration, Electrophysiological evaluation, Inflammation, Histology

## Abstract

This study evaluated the efficacy of tubular constructs containing stem cells and Type I collagen, both independently and in conjunction with low-level laser therapy (LLLT), in repairing the sciatic nerve in a rat model. In this animal study, the right sciatic nerve of 30 male Wistar rats, each weighing 250–300 g, was surgically excised to a length of 8 mm. The rats were then randomly allocated to three groups (n = 10 per group). In Group 1, the excised nerve segment was utilized as an autograft and sutured at the defect site. In Group 2, a tubular construct containing stem cells and Type I collagen was used to bridge the proximal and distal ends of the nerve. Group 3 received the same intervention as group 2, supplemented with 5 weeks of LLLT. After 5 and 12 weeks, rats underwent histological, behavioral, and electrophysiological assessments. Data were statistically analyzed using analysis of variance (ANOVA), Bonferroni post-hoc test, and Kruskal–Wallis test. At both 5 and 12 weeks, axonal count and nerve repair scores showed no significant differences among the three groups (*P* > 0.05). Notably, the Sciatic Functional Index (SFI) was the most favorable (lowest) in the autograft group, whereas the stem cell-only group exhibited the least favorable (highest) SFI at 5 weeks (*P* < 0.001). Additionally, distal latency was highest in the stem cell group and lowest in the stem cell combined with LLLT group at 5 weeks (*P* < 0.001). A significant difference was observed between the autograft and stem cell plus LLLT groups (*P* < 0.05). In conclusion, the application of stem cell-laden tubular constructs in conjunction with LLLT demonstrated efficacy for sciatic nerve repair in rats.

## Introduction

Peripheral nerve injury is a relatively common complication associated with oral and maxillofacial surgical procedures, and can also occur as a result of facial trauma. In the United States, over 25,000 patients experience traumatic peripheral nerve injuries annually, with only 15% deemed curable using conventional modalities. If left untreated, these injuries can result in persistent pain, paresthesia, reduced muscle strength, and significant impairment of daily activities [1]. The tension-free suturing technique of nerve ends was first described in 1873 [2]. Evidence indicates that even 10% tension at the suture sites can reduce microvascular blood flow by up to 50%. Additionally, the suturing process may lead to the degeneration of some nerve fibers in the approximation of the two nerve ends, resulting in approximately 50% of sensory and motor neurons not being adequately targeted [3]. The risk of peripheral nerve injury is particularly high in the maxillofacial region because of the dense network of peripheral nerves and the frequency of various oral and maxillofacial surgical procedures. For instance, the risk of lingual nerve damage during surgical extraction of third molars is approximately 0.5% [4]. Moreover, the incidence of inferior alveolar nerve injury during sagittal osteotomy of the mandible ranges from 3.1 to 7% [5], and facial nerve damage occurs in approximately 0.1% of such procedures [6]. Autografts are considered the gold standard for nerve grafting owing to their excellent biocompatibility, low toxicity, and ability to enhance cell migration and attachment. Autografting is the preferred treatment for peripheral nerve injuries [7]. However, autografts have certain limitations, including limited availability, donor site morbidity, and the need for an additional surgical procedure to harvest the graft [8]. Additionally, there may be discrepancies between the harvested nerve and recipient site. Consequently, researchers are actively exploring novel techniques for improving the quality of nerve grafting.

A proposed novel approach to regenerate injured nerves involves employing surgical techniques that utilize growth factors and various cell types, particularly mesenchymal stem cells (MSCs) [7, 9]. MSCs, which can be derived from multiple sources, are multipotent cells capable of differentiating into neural cells and secreting neurotrophic factors that facilitate nerve repair and regeneration [9]. Studies have demonstrated that MSCs play a critical role in promoting the regeneration of the neurovascular barrier and clearing myelin debris, which are both essential processes for effective nerve healing [10]. When combined with scaffolds such as acellular nerve allografts (ANA), MSCs provide structural support along with immune-modulating benefits, significantly enhancing repair outcomes [9, 10]. Allografts or vascularized nerve grafts are recommended in cases in which extensive nerve grafting is necessary. Allografts act as temporary scaffolds for nerve regeneration; however, their use typically requires immunosuppressive therapy such as FK506 [11]. Another alternative to autografts is the use of nerve-guiding tubes made from synthetic materials such as silicone [12]. While silicone tubes have certain drawbacks, including eliciting an immune response and the need for secondary surgery for tube removal, they have demonstrated acceptable results in the repair of nerve defects [13, 14]. However, despite significant advancements, these artificially synthesized alternatives generally perform worse than traditional nerve grafting techniques [7]. To enhance the quality of nerve regeneration and repair, growth-promoting factors, stem cells, and medications such as citicoline may be utilized [15]. Evidence indicates that the tube technique is not effective for defects larger than 3 cm, often yielding unfavorable outcomes [16]. Collagen can be used to fill gaps within the tube, prevent collapse, and improve the quality of nerve repair [17]. Moreover, previous studies have shown that the incorporation of stem cells can enhance nerve growth in vitro and promote axonal repair in various animal models [7]. MSC-supplemented scaffolds, such as acellular nerve allografts (ANAs), have shown significant potential for repairing segmental nerve defects by integrating structural support with the biological and immunomodulatory properties of MSCs [9, 10]. However, concerns regarding the immune response to allograft transplantation as well as to non-autologous stem cell transplants persist. Consequently, the immune system is typically suppressed by medication when these transplantation techniques are employed [7]. Low-level laser therapy (LLLT) has emerged as a promising approach to enhance nerve regeneration and repair. LLLT can reduce scarring, promote axonal growth and myelination, minimize the migration of mononuclear cells (thereby decreasing edema), and facilitate healing via its anti-inflammatory effects [18]. Nevertheless, additional studies are necessary to establish the optimal efficacy of LLLT in nerve repair. Given the insights from previous research and the existing knowledge gap on this subject, this study aimed to evaluate the efficacy of tubes containing stem cells and type I collagen, both alone and in combination with LLLT, for sciatic nerve repair in rat models.

## Method and materials

This animal study was conducted using 30 male Wistar rats obtained from the Pharmacological Science Center of Tehran University of Medical Sciences. All procedures adhered to the established guidelines for the care and use of laboratory animals and were approved by the Ethics Committee of the School of Dentistry at Islamic Azad University, Tehran (IR.IAU.DENTAL.REC.1400.135).

### Intervention

The rats used in this study were 8–10 weeks old, weighing between 250 and300 g. They were housed in cages for a two-week acclimation period with ad libitum access to food and water to reduce stress. On the day of surgery, anesthesia was induced by administering 90 mg/kg ketamine (Kepro, Deventer, Netherlands) and 12.5 mg/kg of xylazine (Rampon, Bayer, Germany) via intraperitoneal injection [19].

Following anesthesia, the right leg of each rat was shaved and a cutaneous incision was made on the posterolateral surface of the thigh. The right sciatic nerve was excised over an 8 mm segment between the popliteal cavity and the ischial spine. The rats were randomly assigned to three groups (n = 10 each).

**Fig. 1:**
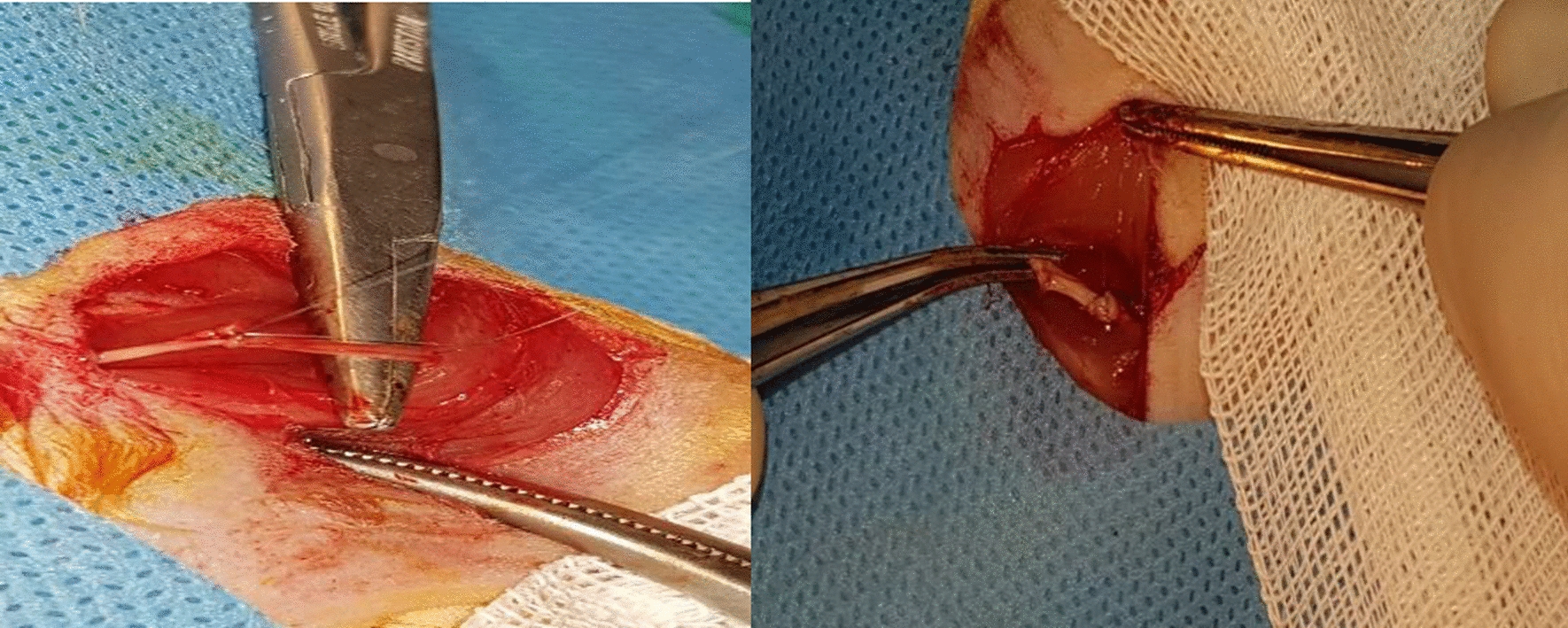
Autograft suturing in the control group

**Fig. 2:**
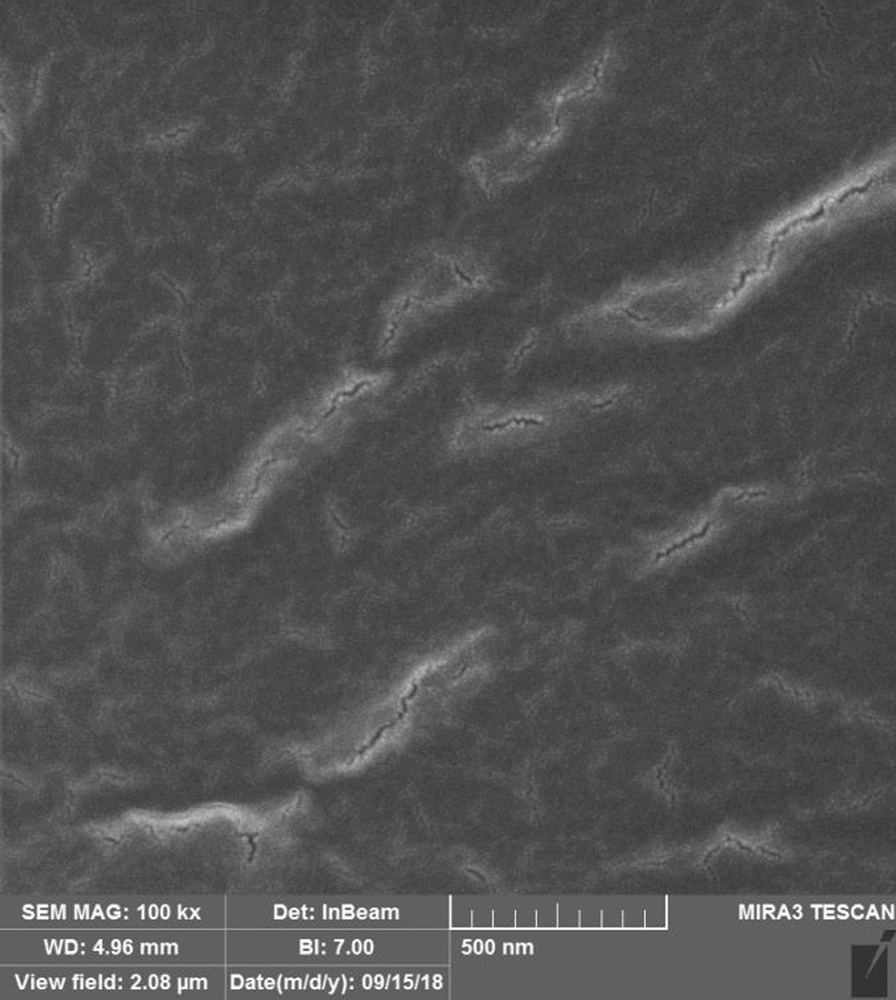
Scanning electron microscopic micrograph of a tube cross-section containing type I collagen without dental pulp stem cells at 100,000 × magnification

**Fig. 3:**
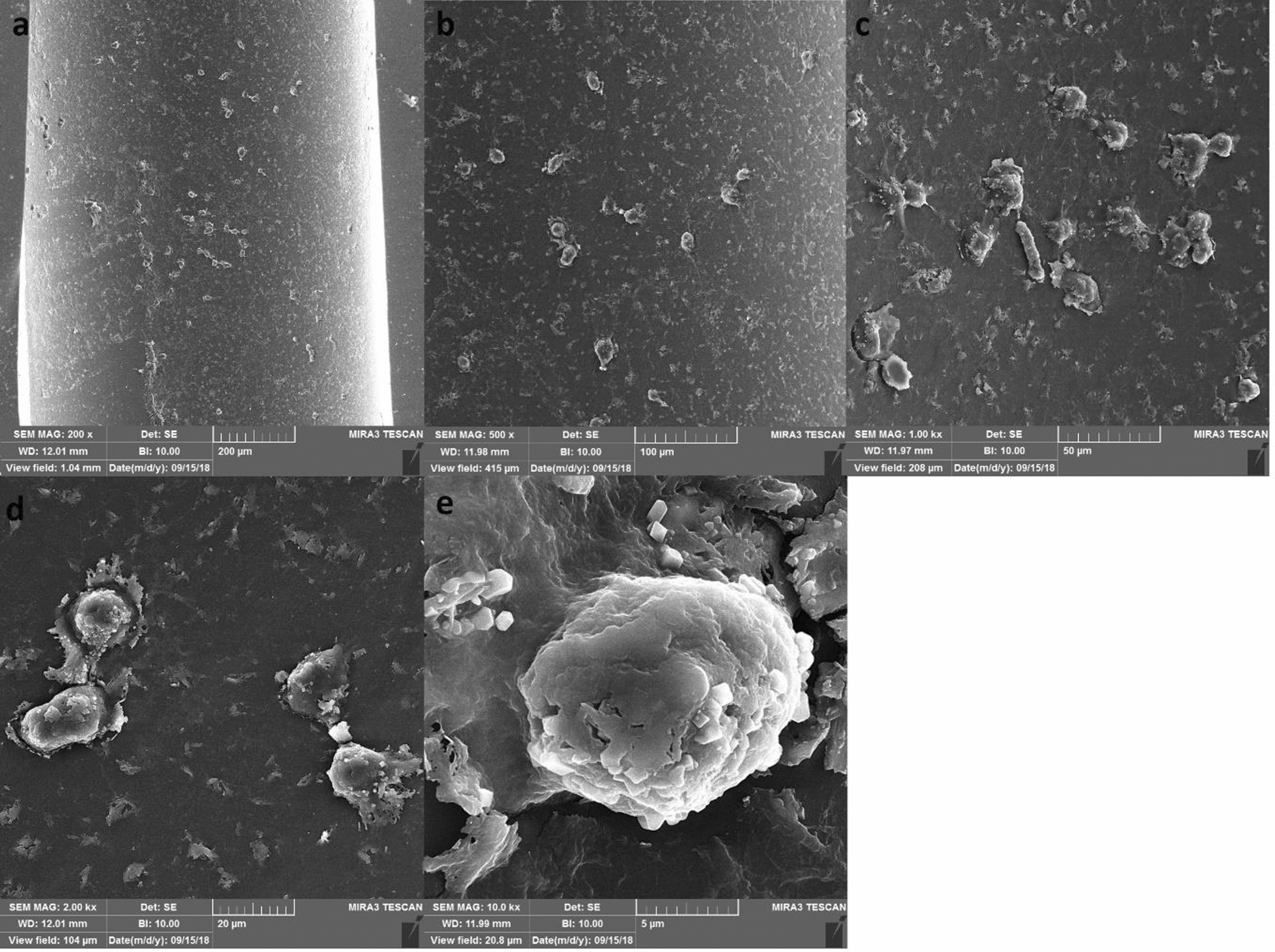
Scanning electron microscopic micrograph of a tube cross-sections containing type I collagen and dental pulp stem cells at 200x **a**, 500x **b**, 1000x **c**, 2000x **d**, 5000x **e**, 10,000x **f** magnification

In the control group, the excised nerve was autografted at the defect site and securely sutured using 0–9 nylon sutures (Fig. 1). In the first experimental group, a silicone tube containing stem cells (Binasaz, Tehran, Iran) was used. This tube was filled with type I collagen (BD BioScience) and human dental pulp stem cells obtained from the Iranian Genetic Research Center. The surface markers of these cells have been previously characterized, confirming their osteogenic and adipogenic properties. Following the seeding of stem cells into the tubes, their cross-sections were analyzed using a scanning electron microscope (Figs. 2 and 3).

**Fig. 4:**
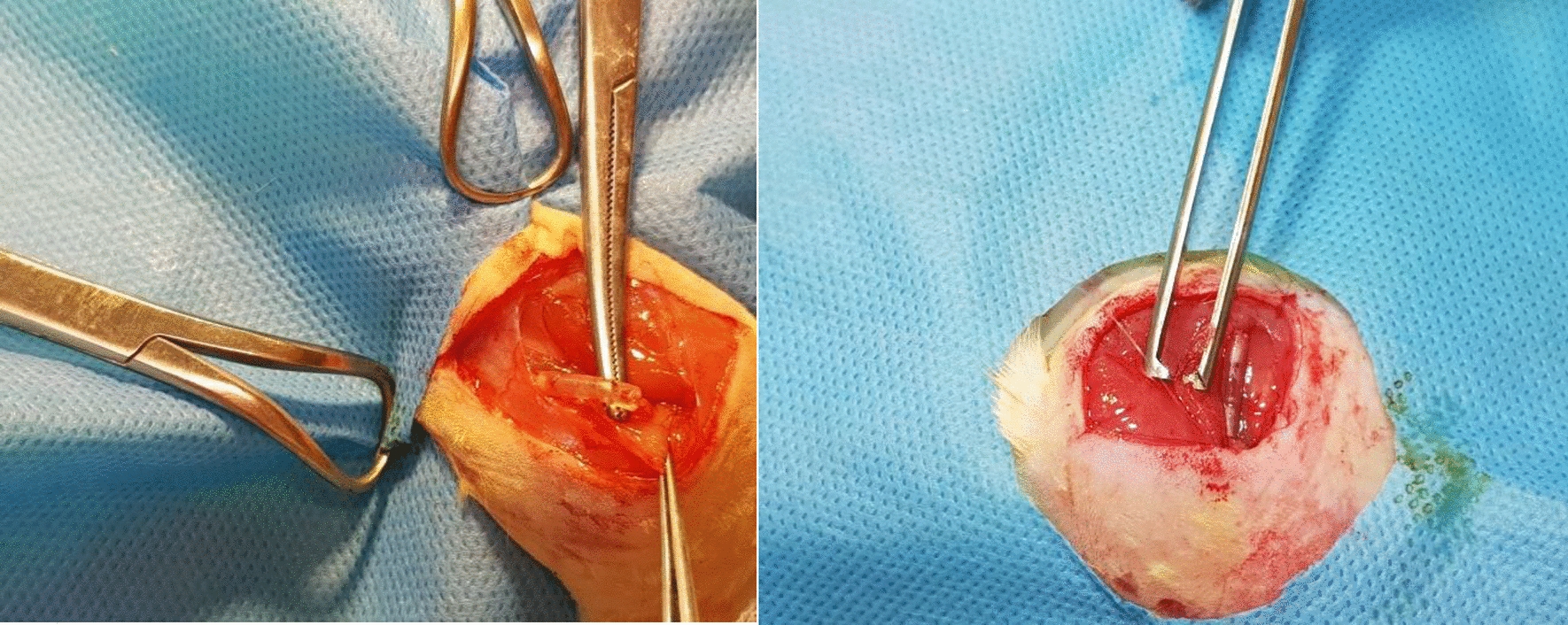
Grafting of silicone tube containing type I collagen and stem cells in the first experimental group

The silicone tube used in the first experimental group had an internal diameter of 2 mm, allowing for insertion of 1 mm from the proximal and distal ends of the excised nerve. These nerve ends were sutured to the internal wall of the tube using nylon sutures (Fig. 4).

In the second experimental group, the same silicone tube was grafted at the defect site as in the first group, but with an important difference; these rats received low-level laser therapy (LLLT) three times a week for a total duration of five weeks. LLLT was administered using a red laser at a wavelength of 660 nm (Azor, Russia) [20] in continuous pulse mode with an energy density of 1 J/cm^2^ and a power output of 50 mW. The laser was applied in contact mode along the injured nerve path for 20 s during each session [18]. The probe tip was 1 cm long.

All surgical procedures were performed by the same veterinarian to ensure consistency. After surgery, the rats were housed in cages with ad libitum access to food and water and maintained under a 12-h light/dark cycle for 8 weeks [21]. Additionally, all rats received daily injections of cyclosporine A at a dosage of 1.5 mg per 100 g body weight for 2 weeks [7].

### Behavioral analysis

After the surgical procedures, behavioral assessments were conducted on the rats to evaluate functional recovery. Five randomly selected rats from each group were assessed 5 weeks post-surgery, while the remaining five rats were evaluated 12 weeks post-surgery. The sciatic functional index (SFI) was used to measure the extent of recovery of sciatic nerve function [22]. In addition to the SFI, locomotor functional capacity was evaluated using the Basso, Beattie, and Bresnahan (BBB) scale [23]. This scale was designed to assess locomotor performance and recovery in rats after nerve injury.

### SFI assessment

For the assessment of the sciatic functional index (SFI), the soles of the rats were first coated in ink. Subsequently, the rats were placed in a wooden cylinder measuring 130 × 25 × 25 cm to encourage them to crawl across a specially designed pad. Footprints were collected, and several measurements were taken from the prints:

- Sole length,
- Distance between the 1st and 5th toes,
- Distance between the 2nd and 4th toes.

These measurements were recorded in millimeters and then used in the following formula to calculate the SFI [20].

The formula used to calculate the sciatic functional index (SFI) is as follows:

$${\text{SFI }} = - 38.3\left( {{\text{PLF}}} \right) + 109.5\left( {{\text{TSF}}} \right) + 13.3\left( {{\text{ITSF}}} \right) - 8.8$$

$$\text{PLF}=\frac{EPL-NPL}{NPL}$$

$$\text{TSF}=\frac{ETS-NTS}{NTS}$$

$$\text{ITSF}=\frac{EITS-NITS}{NITS}$$

where E is the experimental side, N is the normal side, PL is the print length (distance between the heel and the third toe), TS is the toe spread (distance between the tip of the 1st and the 5th toes), and ITS is the intermediate toe spread (distance between the 2nd and 4th toes).

A score of − 100 indicates complete dysfunction, while a score of 0 indicates normal function.

### BBB scale

The rats were placed in a plastic cylinder with a diameter of 106.5 cm and a height of 60 cm for locomotor assessment. They were videotaped during this evaluation. In this scoring system, a score of 0 indicated no observable movement of the hindlimbs, while a score of 14 represented complete support of weight. The maximum score of 21 indicated normal hindlimb function [24]. Table 1 presents the detailed scoring system of the BBB scale [23, 24].

**Table 1:** Basso, beattie, bresnahan (BBB) functional scale for assessment of the locomotor capacity of rats after spinal cord injury

0	No observable movement of the hindlimbs
1	Slight (limited) movement of one or two joints, usually hip and/or knee
2	Extensive movement of one joint or extensive movement of one joint and slight movement of the other
3	Extensive movement of two joints
4	Slight movement of all three joints of the hindlimbs
5	Slight movement of two joints and extensive movement of the third joint
6	Extensive movement of two joints and slight movement of the third joint
7	Extensive movement of the three joints in the hindlimbs
8	Sweeping without weight bearing or plantar support of the paw without weight bearing
9	Plantar support of the paw with weight bearing only in the support stage (i.e., when static) or occasional, frequent or inconsistent dorsal stepping with weight bearing and no plantar stepping
10	Plantar stepping with occasional weight bearing and no forelimb-hindlimb coordination
11	Plantar stepping with frequent to consistent weight bearing and no forelimb-hindlimb coordination
12	Plantar stepping with frequent to consistent weight bearing and occasional forelimb- hindlimb coordination
13	Plantar stepping with frequent to consistent weight bearing and frequent forelimb-hindlimb coordination
14	Plantar stepping with consistent weight support, consistent forelimb-hindlimb coordination and predominantly rotated paw position (internally or externally) during locomotion both at the instant of initial contact with the surface as well as before moving the toes at the end of the support stage or frequent plantar stepping, consistent forelimb-hindlimb coordination and occasional dorsal stepping
15	Consistent plantar stepping, consistent forelimb-hindlimb coordination and no movement of the toes or occasional movement during forward movement of limb; predominant paw position is parallel to the body at the time of initial contact
16	Consistent plantar stepping and forelimb-hindlimb coordination during gait and movement of the toes occurs frequently during forward movement of the limb; the predominant paw position is parallel to the body at the time of initial contact and curved at the instant of movement
17	Consistent plantar stepping and forelimb-hindlimb coordination during gait and movement of the toes occurs frequently during forward movement of limb; the predominant paw position is parallel to the body at the time of initial contact and at the instant of movement of the toes
18	Consistent plantar stepping and forelimb-hindlimb coordination during gait and movement of the toes occurs consistently during forward movement of limb; the predominant paw position is parallel to the body at the time of initial contact and curved during movement of the toes
19	Consistent plantar stepping and forelimb-hindlimb coordination during gait and movement of the toes occurs consistently during forward movement of limb; the predominant paw position is parallel to the body at the instant of contact and at the time of movement of the toes, and the animal presents a downward tail some or all of the time
20	Consistent plantar stepping and forelimb-hindlimb coordination during gait and movement of the toes occurs consistently during forward movement of limb; the predominant paw position is parallel to the body at the instant of contact and at the time of movement of toes, and the animal presents consistent elevation of the tail and trunk instability
21	Consistent plantar stepping and coordinated gait, consistent movement of the toes; paw position is predominantly parallel to the body during the whole support stage; consistent trunk stability; consistent tail elevation

### Electrophysiological analysis

At 5 and 12 weeks post-surgery, the sciatic nerves of the rats were exposed under anesthesia. Measurements of distal latency and amplitude were recorded after electrical stimulation was applied to the proximal site of the injured nerve. The compound muscle action potential was recorded from the gastrocnemius muscle using a needle electrode, with a reference cap electrode positioned at the knee joint. To ensure accurate readings, a stainless steel needle was inserted into the skin of the tail to serve as a ground electrode [24].

### Histological analysis

**Fig. 5:**
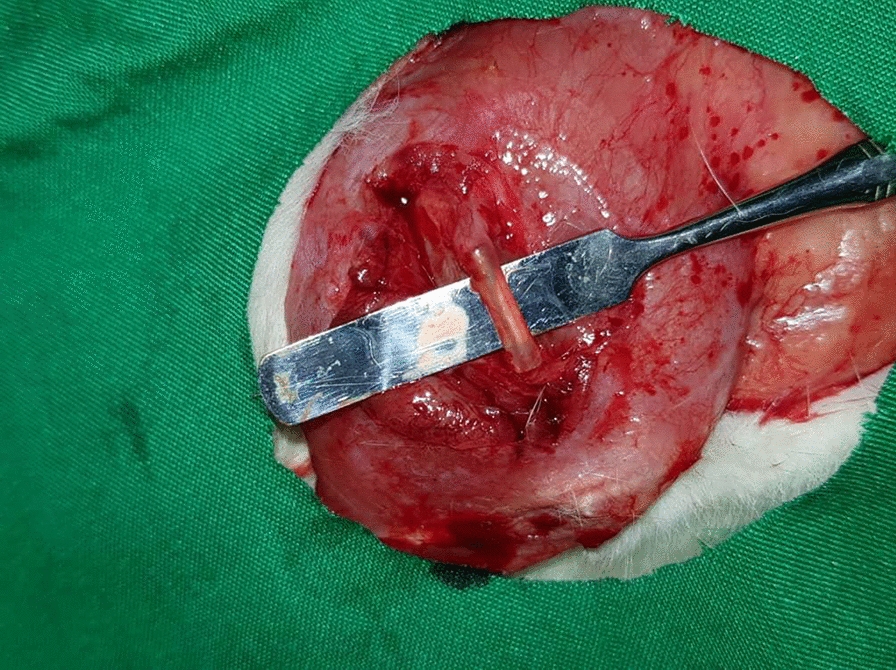
Exposure of the nerve repaired with silicone tube containing collagen and stem cells

At 5 and 12 weeks, the rats were sacrificed through an overdose of anesthetic agent. The repaired nerve was exposed (see Fig. 5) and carefully dissected for histological analysis. The proximal nerve end was marked, and the nerve segment was immersed in 10% formalin for preservation.

Three sections were prepared from each specimen:

1. A transverse section at the proximal nerve end,
2. A transverse section at the distal nerve end,
3. A longitudinal section.

The sections were stained with hematoxylin and eosin (H&E) for visualization.

The following assessments were conducted.

- The number of axons was counted in the transverse sections.
- The contents of the nerve tubes were evaluated in longitudinal sections.

The following parameters were scored as shown in Table 2.

- Continuity of axons,
- Activity of fibroblasts,
- Amount of collagen,
- Quality of myelin sheaths,
- Number of inflammatory cells,
- Status of the vascular network.

**Table 2:** Scoring of histological parameters

Category	Subcategory	Score
Continuity of axonal Fibers between proximal and distal ends	Weak or unorganized proliferation of axons	+ 1
	Moderate proliferation of axons	+ 2
	Ongoing regeneration in the treated area	+ 3
Collagen synthesis by fibroblasts	Dense	+ 1
	Moderate	+ 2
	Normal	+ 3
Myelin sheaths	None or sparsely formed	+ 1
	Cavitated and weak	+ 2
	Circular and homogeneous	+ 3
Inflammatory cells (× 40)	≥ 10 cells	+ 1
	1–9 cell(s)	+ 2
	No inflammatory cells	+ 3
Vasculature (× 20)	≥ 6	+ 1
	3–6	+ 2
	1–3	+ 3

The degree of nerve repair was calculated using the following five indices:

1. Axonal continuity,
2. Frequency of fibroblast and collagen synthesis,
3. Quality of myelin sheaths,
4. Infiltration of inflammatory cells,
5. Status of the vascular network.

**Fig. 6:**
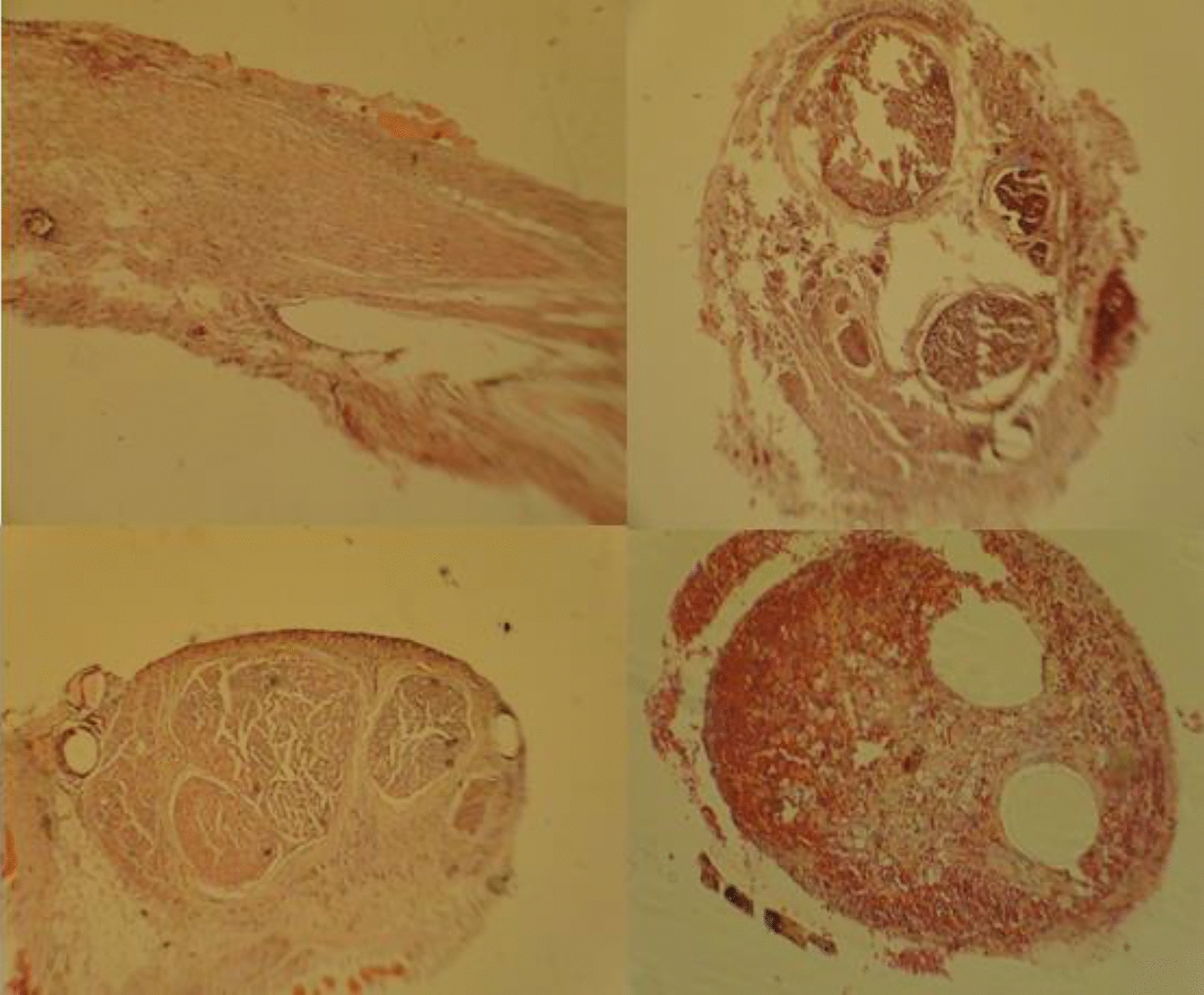
Histological section of repaired nerves; upper right: transverse section of nerve repaired with silicone tube at 5 weeks; upper left: longitudinal section of the nerve repaired with silicone tube and LLLT at 5 weeks; lower right: transverse section of the nerve repaired with silicone tubes at 12 weeks; lower left: transverse section of the nerve repaired by autografting at 12 weeks (control group) (H & E, 4 × magnification)

Each specimen was assigned a total score:

- Scores 0 to 5 indicated poor repair,
- Scores 6 to 10 indicated incomplete repair,
- Scores 11 to 15 indicated complete repair (see Fig. 6) [24].

### Statistical analysis

Repair scores were compared among the three groups using statistical methods. For quantitative variables, analysis was conducted using ANOVA, followed by a post-hoc Bonferroni test. The Kruskal–Wallis test was used for qualitative variables. The significance level was set at 0.05.

## Results

During the study, one rat from the autograft group died at five weeks, and one rat from the first experimental group expired post-surgery.

Table 3 presents the results of the six evaluated variables: axonal number, nerve repair score, BBB scale, SFI, distal latency, and amplitude across the three groups at 5 and 12 weeks.

**Table 3:** Results regarding the six variables (axonal number, nerve repair score, BBB scale, SFI, distal latency, and amplitude) evaluated in the three groups at 5 and 12 weeks

Group/parameter	Assessment time point	Axonal number	Degree of nerve repair	BBB scale	SFI	Distal latency	Amplitude
Autografting	5 weeks	136.5 ± 19.70	11.50 ± 1.00	14.25 ± 1.08	16.29 ± 0.83	1.87 ± 0.25	2660.4 ± 4976.4
	12 weeks	231.0 ± 7.97	8.1 ± 11.60	15.80 ± 1.16	12.48 ± 1.32	1.27 ± 0.22	4300.0 ± 1211.6
Silicone tube alone	5 weeks	144.20 ± 20.2	10.75 ± 0.82	13.75 ± 0.43	32.87 ± 0.87	2.00 ± 0.36	3006.5 ± 3283.5
	12 weeks	230.00 ± 7.23	11.80 ± 0.74	15.60 ± 1.01	14.90 ± 1.17	1.25 ± 0.34	3578.0 ± 791.25
Silicone tube plus LLLT	5 weeks	133.00 ± 16.07	12.80 ± 1.16	14.60 ± 0.80	18.25 ± 1.00	1.24 ± 0.23	4362.4 ± 1570.4
	12 weeks	239.4 ± 19.9	12.00 ± 1.16	15.60 ± 0.80	12.78 ± 0.69	1.60 ± 0.06	4630.0 ± 1083.4

*Axonal number*

The axonal number was nearly identical across all three groups at both 5 (*P* = 0.2) and 12 weeks (*P* = 0.2).

*Degree of nerve repair*

There were no significant differences in the degree of nerve repair among the three groups at 5 weeks (*P* = 0.6) or 12 weeks (*P* = 0.7).

*BBB scale*

The BBB scale did not show significant differences among the three groups at 5 weeks (*P* = 0.8) or 12 weeks (*P* = 0.8).

*SFI*

At 5 weeks, SFI was the lowest (most favorable) in the autograft group and the highest (least favorable) in the silicone tube group. The differences between the three groups were significant (*P* < 0.001). Pairwise comparisons indicated a significant difference between the autograft and silicone tube groups (*P* < 0.001), whereas no other significant differences were noted (*P* > 0.05). At 12 weeks, there were no significant differences in SFI among the three groups (*P* = 0.2).

*Distal latency*

At 5 weeks, the maximum distal latency was observed in the silicone tube group, whereas the minimum was observed in the silicone tube plus LLLT group. The difference among the three groups was significant (*P* < 0.001). Pairwise comparisons revealed a significant difference only between the autograft and silicone tube plus laser groups (*P* < 0.05), with no other significant differences noted (*P* > 0.05).

At 12 weeks, distal latency showed no significant differences among the groups (*P* = 0.2).

*Amplitude*

The amplitude was consistent across all three groups, with no significant differences at both 5 weeks (*P* = 0.2) and 12 weeks (*P* = 0.2).

Overall, the results indicated significantly greater improvements in axonal number, SFI, and distal latency at 5 weeks than at 12 weeks (*P* < 0.05).

## Discussion

This study assessed the efficacy of tubes containing stem cells and type I collagen, either alone or in combination with low-level laser therapy (LLLT), for sciatic nerve repair in rats. An autologous nerve graft was used as a control. The results indicated that the use of silicone tubes containing type I collagen and stem cells played a pivotal role in nerve repair, yielding results comparable to those of autografting in most evaluated parameters.

A previous study by Mohammadi et al. [25] highlighted that the application of omental adipose-derived nucleated cells (OADNCs) enhances sciatic nerve regeneration, similar to undifferentiated bone marrow stromal cells (BMSCs), suggesting that OADNCs should be further studied in the field of regenerative medicine and surgery. In contrast to their findings, our study employed a different source of stem cells, focusing on human dental pulp stem cells (DPSCs), which are derived from human adult teeth. DPSCs are considered a reliable and accessible source for regenerative therapies, and have shown promise in previous studies.

Our findings contribute to a growing body of evidence supporting the use of stem cells in nerve repair, particularly highlighting the potential of DPSCs as a therapeutic option. Comparison with autologous grafts strengthens the argument for exploring alternative sources of stem cells in regenerative medicine.

Another major difference between the two studies is in the conduits used to bridge the gap between the transected sciatic nerve stumps. Mohammadi et al. employed a vein conduit that provided a scaffold for nerve regeneration. Vein grafts were inserted into the proximal and distal nerve stumps to facilitate nerve fiber growth and myelination. In contrast, our study utilized a silicone tube as a conduit, which offers a biocompatible scaffold and allows better control over the microenvironment of the graft site. Furthermore, our study introduces low-level laser therapy (LLLT) as a novel therapeutic approach alongside stem cell implantation. LLLT has been documented to promote nerve regeneration through various mechanisms, including reduction of inflammation, increased cell proliferation, and enhanced expression of nerve growth factors. This innovative addition underscores the potential of combining therapeutic strategies to improve the nerve repair outcomes.

Our study expanded upon the methodologies of Mohammadi et al. by incorporating a comprehensive range of assessments: behavioral, electrophysiological, functional, and histological assessments. The inclusion of electrophysiological testing provided a more thorough evaluation of nerve function, offering critical insights into the nerve’s ability to transmit electrical signals, which is essential for functional recovery. This combination of assessments allows for a more holistic understanding of the regenerative potential of stem cell therapy and LLLT, moving beyond mere structural analyses to encode functional implications.

While Mohammadi et al.’s study concentrated primarily on functional recovery through walking track analysis (SFI) and histological assessments, our approach aimed to provide a broader understanding of the mechanisms and outcomes of nerve repair interventions.

Both studies reported the positive effects of stem cell therapy on nerve regeneration. However, the inclusion of low-level laser therapy (LLLT) alongside stem cells in our study may have enhanced the regenerative outcomes compared with the findings of Mohammadi et al. Specifically, LLLT may have modulated inflammation and created a more favorable microenvironment for nerve regeneration, contributing to improved functional recovery.

In a related study by Taifebagerlu et al. [26], local application of Brain-Derived Neurotrophic Factor (BDNF) was demonstrated to accelerate functional recovery after transection of the sciatic nerve. Notably, both our study and that of Taifebagerlu et al. utilized silicone tube conduits to bridge the gap between the transected sciatic nerve stumps, allowing for axonal growth along the scaffold provided by silicone.

The key distinction lies in the cellular components of these conduits. In this study, we focused on human dental pulp stem cells (DPSCs) to promote nerve regeneration. DPSCs are known for their regenerative properties and their potential for nerve regeneration. Conversely, Taifebagerlu et al. employed BDNF, a neurotrophin recognized for its crucial role in enhancing axonal regeneration and promoting the migration of Schwann cells, along with providing neuroprotection.

This difference in regenerative strategies underscores the diverse potential of stem cell therapy and neurotrophic factors in enhancing nerve repair, and suggests that a combination approach incorporating both DPSCs and BDNF, alongside LLLT, might yield further beneficial outcomes.

In contrast to the approach adopted by Taifebagerlu et al., our study evaluated the effects of low-level laser therapy (LLLT) as a novel adjunct to stem cell therapy. This addition allowed us to investigate not only the regenerative potential of human dental pulp stem cells (DPSCs) but also how LLLT influences inflammation and nerve regeneration. LLLT has been reported to exert neuroprotective effects and to facilitate regeneration through mechanisms such as reducing inflammation and promoting cellular repair. This unique aspect of our approach enabled us to assess the combined effects of DPSCs and LLLT in a more holistic manner by evaluating both functional and histological outcomes.

Both studies utilized behavioral tests to assess functional recovery, with Taifebagerlu et al. employing the Basso, Beattie, and Bresnahan (BBB) locomotor rating scale, Sciatic Functional Index (SFI), and Static Sciatic Index (SSI) to evaluate motor function. In contrast, our study placed greater emphasis on electrophysiological tests, such as Compound Muscle Action Potential (CMAP) and nerve conduction velocity (NCV). These tests provide a more direct measurement of nerve function at the cellular level. Including electrophysiological assessments in our study allowed for a more comprehensive evaluation of nerve regeneration, complementing behavioral and histological analyses.

Our multidimensional approach underscores the importance of evaluating outcomes from various perspectives (functional, histological, and electrophysiological) to gain a deeper understanding of the therapeutic potential of stem cells and LLLT in nerve regeneration. By integrating these different modalities of assessment, we can more effectively elucidate the mechanisms underlying regeneration and overall efficacy of combined therapies.

Kolar et al. [7] demonstrated that dental pulp stem cells (DPSCs) exhibit optimal efficacy for nerve repair, showing promise in both in vitro experiments and animal models. They highlighted the ability of DPSCs to produce various mediators that play a crucial role in the repair of peripheral nerves. Although their findings align with the results of the present study, it is important to note that they sourced stem cells from multiple individuals, in contrast to our approach of utilizing DPSCs from a single source.

Sasaki et al. [27] also contributed to the understanding of nerve repair by comparing autografts and silicone tubes and found no significant difference in muscle function and electrophysiological outcomes after 13 weeks of follow-up. Their results corroborated our findings; however, they did not explore the impact of low-level laser therapy (LLLT). Our study emphasizes the significant role of LLLT in enhancing the healing process and provides additional insights into improving the regenerative outcomes.

In their earlier research, Sasaki et al. [28] investigated the efficacy of using rat dental pulp stem cells for peripheral nerve repair and reported that this intervention led to improvements in axonal thickness and myelin quality. The present study supports these findings, demonstrating comparable quality of myelin sheaths and axonal counts in both the experimental groups and autograft control groups. This suggests that LLLT may further enhance the therapeutic effects of DPSCs in nerve regeneration.

Buchaim et al. [18] established the optimal efficacy of low-level laser therapy (LLLT) for peripheral nerve repair, providing compelling evidence through both histological and morphological assessments. Similarly, Zacarra et al. [29] explored the in vitro effects of LLLT on human dental pulp stem cells (DPSCs), demonstrating significant impacts on cell proliferation and viability. However, their findings diverge from those of the present study.

In our study, there were no significant differences in the histological parameters and axonal counts between the silicone tube groups with and without LLLT. The sole distinction observed was in the inflammation index, suggesting that, while LLLT may modulate inflammatory responses, it did not significantly enhance overall histological outcomes or axonal regeneration in this context.

The discrepancies between the findings of Zacarra et al. and our results may be attributed to variations in the laser parameters, including wavelength, intensity, and treatment duration. These factors can significantly influence biological responses elicited by LLLT, leading to different outcomes.

In summary, the present study demonstrated that the outcomes of employing a silicone tube containing dental pulp stem cells (DPSCs) along with low-level laser therapy (LLLT) were comparable to those achieved through standard autografting at both 5 and 12 weeks. This combination technique may serve as a viable alternative to autografting, provided that certain considerations are taken into account.

Histological analysis revealed no significant differences in key parameters between the silicone tube plus LLLT and the autograft groups at both time points, corroborating the findings of Sasaki et al. [28]. The only notable distinction in histological parameters was the marked superiority of the silicone tube plus LLLT group in terms of the inflammation index at 5 weeks, consistent with the observations made by Buchaim et al. [18].

Inflammation surrounding the silicone tube is a known complication associated with its implantation. Notably, the degree of inflammation was significantly reduced within the LLLT group compared to the silicone tube group without laser intervention. This suggests that LLLT may mitigate inflammatory responses, enhance biocompatibility, and potentially improve the overall healing outcomes in peripheral nerve repair.

In the present study, the three groups exhibited no significant differences in axonal counts at either 5 or 12 weeks, suggesting that the interventions tested displayed optimal efficacy compared with autografting, which remains the gold standard for nerve repair. However, contrasting results were observed by Zacarra et al. [29], who found no differences between laser and non-laser groups in their animal models.

Regarding the functional assessment captured by the Sciatic Function Index (SFI), the autograft group demonstrated significantly improved function compared with the silicone tube group. Notably, the silicone tube group also outperformed the laser group at 5 weeks. However, no significant differences emerged among the groups at the 12-week mark, indicating that optimal functional recovery may require an extended timeframe in the non-laser groups.

In line with these findings, Yang et al. [20] asserted that stem cells alone may not significantly accelerate functional recovery. Nevertheless, the combination of stem cells and LLLT appeared to markedly accelerate recovery processes. In contrast to the SFI results, the three groups showed no significant differences in the Basso-Beattie-Bresnahan (BBB) scale at either 5 or 12 weeks.

This discrepancy may highlight the different aspects of nerve repair and recovery captured by these assessment tools, emphasizing the need for diverse evaluation methods when considering the functional outcomes of peripheral nerve regeneration.

The electrophysiological assessment of distal latency at 5 weeks demonstrated significantly superior results in the laser group compared to the other two groups, suggesting enhanced attachment between the two ends of the nerve defect, and thus a higher velocity of pulse transmission. However, this difference was not maintained at the 12-week follow-up. These findings align with those regarding SFI, supporting the assertion made by Yang et al. [20] that low-level laser therapy (LLLT) can significantly enhance nerve repair.

Evaluation of the amplitude at both 5 and 12 weeks revealed the best outcomes in the silicone tube plus laser group, indicative of a greater number of nerve fibers present. Similar findings have been reported by Sasaki et al. (27), reinforcing the positive impact of this combinatorial approach on nerve regeneration.

To further elucidate these effects, additional studies involving larger animal models and varying laser parameters are warranted to determine the most effective laser protocols for optimizing nerve repair outcomes.

## Conclusion

In summary, the combination of a silicone tube conduit infused with collagen type I and dental pulp stem cells, supplemented with low-level laser therapy (LLLT), exhibited promising potential for sciatic nerve repair in rat models. Encouraging functional, electrophysiological, and histological outcomes were comparable to those observed in the autografting group. These findings suggest that this innovative approach may serve as a viable alternative for peripheral nerve regeneration.

### Recommendations

The aim of our study was to show the effect of stem cells and laser on nerve repair. In the next step, a study can be designed to investigate the effect of different laser wavelengths. We also recommend the use of more specific staining methods, like S100 that highlights nerve cells specifically in future studies.

### Limitation

A more specific staining method for nerve cells would have been ideal. For example S100 staining method that highlights nerve cells specifically could have enhanced our analysis.

## Data Availability

All data and materials are provided upon request of the journal
